# Sex bias of females in survival from cancer and infections. Is X the answer?

**DOI:** 10.1038/s41416-020-01245-1

**Published:** 2021-01-19

**Authors:** Abdullah Al Emran, Stuart J. Gallagher, Jessamy C. Tiffen, Peter Hersey

**Affiliations:** Melanoma Oncology and Immunology Program, The Centenary Institute, University of Sydney, Royal Prince Alfred Hospital, Missenden Road, Camperdown, NSW 2050 Australia

**Keywords:** Cancer epigenetics, Oncology

## Abstract

Major differences in survival of men and women from infectious diseases and cancers have been highlighted by death rates from COVID-19 infections. In cancer, attention has been focussed on differences in gene expression from X chromosomes in men and women with a preponderance of genes involved in immune responses being expressed in women. Important findings have been that some of the genes are important epigenetic regulators that play fundamental roles in immune responses.

One of the striking features of the coronavirus disease 2019 (COVID-19) outbreak has been the higher death rates in men even though the infection rates seem similar between men and women.^1^ Similar findings were reported from Wuhan where men had 2.4 times the death rate of women^2^ and in New York where press releases stated twice the death rate of men compared with women.^3^ Although men had higher rates of comorbidities, these differences were not considered sufficient to explain the higher death rates and other explanations have been sort. Women are considered to have stronger immune responses against infective diseases and a higher rate of autoimmune diseases, so this has questioned whether the lower death rate may have an immune basis.

A sex bias is not only seen in infections, but also in cancers where a strong sex bias in survival from cancer is well documented.^4,5^ For example, women in Australia have approximately half the death rates from melanoma as males.^6^ A number of explanations have been proposed to account for these major differences in melanoma, such as higher sun exposure in males^7^ and higher mutation rates^8^ in melanoma from males. When stringent statistical analyses are carried out, however, female sex remains as the major contributor to longer survival.^4^

Melanoma is not the only cancer to show improved survival in females and previous researchers have asked whether this may be due to differences in the sex chromosomes between male and females. In a mammoth study, Dunford and colleagues examined information in The Cancer Genome Atlas (TCGA) from 21 different tumour types from 4100 cancers.^5^ They found that 6 out of 783 X chromosome genes had loss-of-function mutations with tumour-suppressive function in males but not in females. There were no similar differences in 18,055 non-X autosomal genes. Importantly, four of the six genes were known epigenetic regulators, such as *KDM6A* (lysine-specific demethylase 6A), *KDM5C* (lysine-specific demethylase 5C), *ATRX* (Alpha thalassaemia/mental retardation syndrome X-linked) and *DDX3X* (DEAD-box helicase 3 X-linked).^5^

These findings point to important differences in X chromosomes between the sexes. The Y chromosome codes mainly for genes that determine male sex, but X chromosomes are quite large and code for >800 genes many of which are involved in immune responses.^9^ To equalise the number of genes between the sexes, one of the X chromosomes in females undergoes inactivation (Xi) of its genes.^10^ The silencing process is, however, not perfect and between 10 and 20% of the genes on the X may be expressed in females depending on the tissue involved. It is probably of significance that failure to silence genes may be particularly high in activated lymphocytes.^11^ As a result of this phenomenon, females have double expression of many genes involved in immune responses compared with males. Biologists have speculated that this is an evolutionary mechanism to protect the species by enhancing immune responses in females against harmful infections.

Analysis of data in the TCGA on 458 melanoma patients revealed that *KDM6A* expression was strongly related to improved survival from melanoma in female patients. *ATRX* had prognostic significance in both sexes. Analysis of another series of 678 patients with earlier melanoma referred to as the Leeds Melanoma Cohort confirmed the association with *KDM6A* expression and also identified *KDM5C* and *DDX3X* as being related to improved survival.^12^ Immune responses are known to be critical in survival from melanoma and the TCGA analysis allowed us to link high *KDM6A* to components of the immune system considered important in killing of melanoma. This was particularly so in the production of interferon γ in female patients which is a key cytokine needed by the immune system to kill cancer cells. Gene set analysis also showed downregulation of Myc and other oncogenic pathways that may have contributed to the improvement in survival.^12^

These data add to a number of studies implicating KDM6A in immune responses against viral infections and in autoimmune diseases.^13^ At a molecular level, KDM6A is known to have an opposing role to EZH2 (enhancer of Zeste homologue 2) in the PRC2 complex in methylation of Lys 27 on H3 histone. This role may explain some of the effects of KDM6A on the immune system in that we previously reported that EZH2 was associated with the repression of several genes associated with antigen presentation and chemokines involved in T cell responses.^14^

Although these studies are compelling in linking KDM6A to immune responses, it is still questionable whether it has a role in immune responses against COVID-19. If this was the case, we would expect that women being treated for severe COVID-19 infections in intensive care would have lower KDM6A expression than those with infections not requiring such care.^15^ We examined the RNA-seq data from blood samples of 102 COVID-19 patients. This included 38 women and 64 men, where 17 women and 34 men were admitted to intensive care unit. The analysis of KDM6A levels in the women showed that treatment in intensive care unit was associated with higher KDM6A expression (GSE157103,^15^ data not shown). Although this was unexpected, it may indicate that KDM6A expression was linked to stronger responses causing higher inflammation in organs such as the lungs. No differences in KDM6A levels were detected in men irrespective of whether they were admitted to intensive care or not.

Female patients with bi-allelic expression of KDM6A may induce the expression of interferon γ pathways which enhance anti-tumour immunity by recruiting immune modulatory cells (Fig. 1). These results point to the need for a better understanding of the role of X-linked genes in immune responses and whether EZH2-mediated suppression of immune modulatory genes have a role in infections as well as in cancer. In cancers and infections that have worst outcomes in males versus females, one approach might be to target (inhibit) the EZH2 epigenetic regulator that opposes KDM6A (Fig. 1). Another option may be to increase levels of KDM6A by administration of oestrogens. Oestrogen α receptors are expressed in practically all lymphocytes and were shown to physically interact with KDM6A to create a permissive chromatin state on endoplasmic reticulum (ER) targets such as C-X-C chemokine motif receptor 4.^16^ It was transactivated by ER to form a feed-forward loop. Administration of 17β-oestradiol has been suggested by others as treatment for COVID-19 infections.^17^

**Fig. 1:**
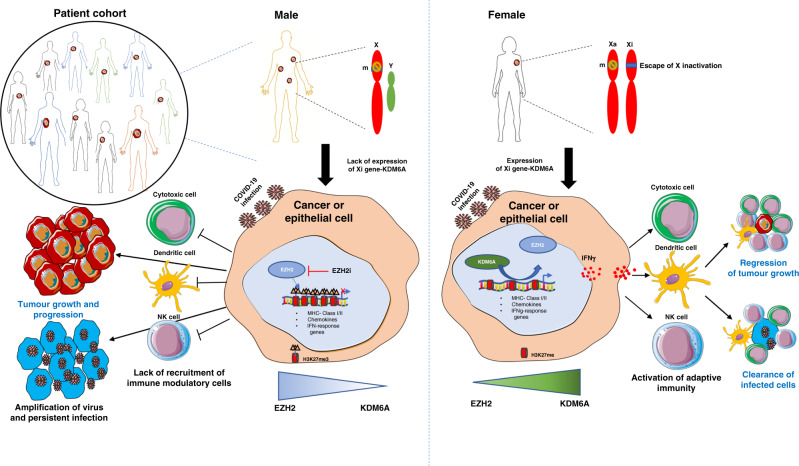
Proposed model of sex-biased role of the X-linked KDM6A gene in promoting immunity. Males harbour one X chromosome with no functional Y chromosome homologue. Hence, mutation in the X-linked epigenetic modifier KDM6A with tumour-suppressive or immunomodulatory role will probably lead to cancer or infection in males. Immune-related genes will be repressed by EZH2-mediated H3K27me3 deposition resulting in low KDM6A protein and immune evasion in male patients. In females, with two X chromosome, [one active (Xa) and one inactive (Xi)], a single mutation (m) in KDM6A is less likely to develop cancer or infections since another functional allele escapes X inactivation. Cells with high KDM6A level would be expected to demethylate H3K27me3 resulting in activation of the interferon γ pathway resulting in inactivation of natural killer (NK), dendritic or cytotoxic T cells to induce anti-tumour immunity and adaptive immunity against virus-infected cells.

These studies have therefore raised many questions that require more detailed study to identify how the powerful survival benefits of the X-linked epigenetic regulators might be used to improve the therapeutic outcome in patients.

## Data Availability

All sources of the publicly available data used in the study are quoted in the commentary.

## References

[1] Wenham C, Smith J, Morgan R (2020). COVID-19: the gendered impacts of the outbreak. Lancet.

[2] Jin, J.-M., Bai, P., He, W., Wu, F., Liu, X.-F., Han, D.-M. et al. Gender differences in patients with COVID-19: focus on severity and mortality. *Front. Public Health* **8**, 152 (2020). 10.3389/fpubh.2020.00152 PMC7201103 32411652

[3] NYC Health. COVID-19: data. https://www1.nyc.gov/site/doh/covid/covid-19-data.page (2020).

[4] Joosse A, Collette S, Suciu S, Nijsten T, Patel PM, Keilholz U (2013). Sex is an independent prognostic indicator for survival and relapse/progression-free survival in metastasized stage III to IV melanoma: a pooled analysis of five European organisation for research and treatment of cancer randomized controlled trials. J. Clin. Oncol..

[5] Dunford A, Weinstock DM, Savova V, Schumacher SE, Cleary JP, Yoda A (2017). Tumor-suppressor genes that escape from X-inactivation contribute to cancer sex bias. Nat. Genet..

[6] Australian Institute of Health and Welfare 2019. *Cancer in Australia 2019*. Cancer series no.119. Cat. no. CAN 123 (AIHW, Canberra, 2019).

[7] Chen J, Shih J, Tran A, Mullane A, Thomas C, Aydin N (2016). Gender-based differences and barriers in skin protection behaviors in melanoma survivors. J. Skin Cancer.

[8] Gupta, S., Artomov, M., Goggins, W., Daly, M. & Tsao, H. Gender disparity and mutation burden in metastatic melanoma. *J. Natl Cancer Inst.* **107**, djv221 (2015). 10.1093/jnci/djv221 PMC4643631 26296643

[9] Libert C, Dejager L, Pinheiro I (2010). The X chromosome in immune functions: when a chromosome makes the difference. Nat. Rev. Immunol..

[10] Colognori D, Sunwoo H, Kriz AJ, Wang C-Y, Lee JT (2019). Xist deletional analysis reveals an interdependency between Xist RNA and polycomb complexes for spreading along the inactive X. Mol. Cell.

[11] Wang J, Syrett CM, Kramer MC, Basu A, Atchison ML, Anguera MC (2016). Unusual maintenance of X chromosome inactivation predisposes female lymphocytes for increased expression from the inactive X. Proc. Natl Acad. Sci. USA.

[12] Emran, A. A., Nsengimana, J., Punnia-Moorthy, G., Schmitz, U., Gallagher, S. J., Newton-Bishop, J. et al. Study of the female sex survival advantage in melanoma-a focus on X-linked epigenetic regulators and immune responses in two cohorts. *Cancers* **12**, 2082 (2020). 10.3390/cancers12082082 PMC7464825 32731355

[13] Itoh Y, Golden LC, Itoh N, Matsukawa MA, Ren E, Tse V (2019). The X-linked histone demethylase Kdm6a in CD4+ T lymphocytes modulates autoimmunity. J. Clin. Investig..

[14] Tiffen, J., Gallagher, S. J., Fabian, F., Dilini, G., Emran, A. A., Cullinane, C. et al. EZH2 cooperates with DNA methylation to downregulate key tumour suppressors and interferon gene signatures in melanoma. *J. Investig. Dermatol.* 140, 2442.e5–2454.e5 (2020). 10.1016/j.jid.2020.02.042 32360600

[15] Overmyer, K. A., Shishkova, E., Miller, I. J., Balnis, J., Bernstein, M. N., Peters-Clarke, T. M. et al. Large-scale multi-omic analysis of COVID-19 severity. *Cell Systems*10.1016/j.cels.2020.10.003 (2020). 10.1016/j.cels.2020.10.003 PMC7543711 33096026

[16] Xie G, Liu X, Zhang Y, Li W, Liu S, Chen Z (2017). UTX promotes hormonally responsive breast carcinogenesis through feed-forward transcription regulation with estrogen receptor. Oncogene.

[17] Breithaupt-Faloppa AC, Correia CJ, Prado CM, Stilhano RS, Ureshino RP, Moreira LFP (2020). 17β-Estradiol, a potential ally to alleviate SARS-CoV-2 infection. Clinics.

